# *Leishmania infantum* infection serosurveillance in stray dogs inhabiting the Madrid community: 2007–2018

**DOI:** 10.1186/s13071-022-05226-6

**Published:** 2022-04-14

**Authors:** Aurora Müller, Ana Montoya, Cristina Escacena, María de la Cruz, Ana Junco, Andrés Iriso, Eloy Marino, Fernando Fúster, Guadalupe Miró

**Affiliations:** 1 Unidad Técnica 6 del Área de Salud Pública, Consejería de Sanidad, Comunidad de Madrid, Madrid, Spain; 2 Departamento de Sanidad Animal, Facultad de Veterinaria, Universidad Complutense, Madrid, Spain; 3 Área de Vigilancia de Riesgos Ambientales en Salud, Consejería de Sanidad, Comunidad de Madrid, Madrid, Spain; 4 Unidad Técnica 1-4-7 del Área de Salud Pública, Consejería de Sanidad, Comunidad de Madrid, Madrid, Spain

**Keywords:** *Leishmania infantum*, Seroprevalence, Stray dogs, Canine leishmaniosis, Risk factors, Zoonosis, Outbreak of human leishmaniosis

## Abstract

**Background:**

Leishmaniosis is an endemic zoonotic disease in the Mediterranean basin caused by *Leishmania infantum* and transmitted by phlebotomine sandflies. While in dogs disease may be severe, leishmaniosis is also a public health concern as was shown in the largest outbreak of human leishmaniosis (HL) in Europe in 2009 occurring in the Madrid region. The aim of the present study was to assess the applicability of the Leishmaniosis Surveillance Program (LeishSP) established in Madrid in 1996 by examining trends in *L. infantum* seroprevalence and associated epidemiological risk factors based on data for the 2007–2018 period.

**Methods:**

The study population consisted of 3225 stray dogs from 17 animal shelters collaborating with the LeishSP. Seroprevalences were recorded twice annually (April and November) from 2007 to 2018. In each yearly period, a minimum of 100 dogs were tested to detect dogs infected before and after the sandfly risk season in Madrid area. Each dog was subjected to the same protocol of blood sample collection and clinical examination to collect epidemiological data and clinical signs. Anti-*Leishmania*-specific IgG was determined by IFAT cut-off ≥ 1:100.

**Results:**

Overall seroprevalence was 6.1% (198 positive dogs). Epidemiological data indicate a significantly higher seroprevalence in dogs > 4 years old, purebred dogs (Pit Bull and related breeds), and medium to large size dogs. There were no seroprevalence differences according to sex and/or season (April and November). In addition, no significant differences were observed according to whether dogs lived inside or outside the HL outbreak area. Remarkably, of 198 dogs testing positive for *L. infantum*, 64.6% had no clinical signs, indicating a high proportion of clinically healthy infected dogs that could be a potential source of infection.

**Conclusions:**

Results indicate a stable seroprevalence of *L. infantum* infection after 2006 in stray dogs in Madrid but with a recent slightly increasing trend. These observations support the need to continue with the LeishSP implemented by sanitary authorities of the Madrid Community as an early warning strategy for human and animal leishmaniosis and to enable continued assessment of the epidemiological role of dogs with subclinical infection in this important zoonotic disease.

**Graphical Abstract:**

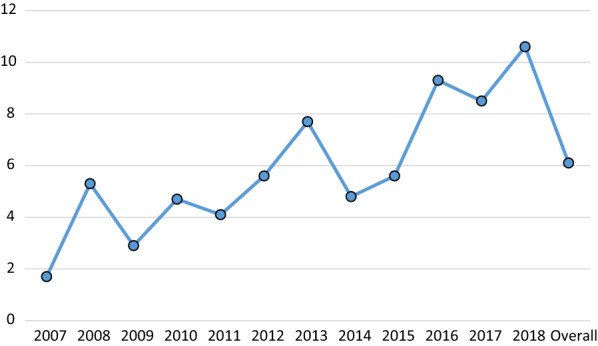

## Background

Leishmaniosis is an endemic zoonotic disease in the Mediterranean basin caused by the parasite *Leishmania infantum* [1]. In Spain, the disease is transmitted by bites of the female sandflies *Phlebotomus perniciosus* and *P. ariasi*. At our latitude, the dog is the main reservoir for the spread of the parasite to humans and other animals [1]. Canine leishmaniosis is a chronic disease with variable clinical signs ranging from subclinical forms seen in clinically healthy infected dogs to other forms presenting with lymphadenomegaly as the most frequent sign, along with exfoliative dermatitis, alopecia, skin ulcers, onychogryphosis, lameness, anorexia, weight loss, cachexia, uveitis, epistaxis, and anaemia, with kidney failure as the most serious clinical manifestation [2, 3]. These clinical manifestations are not always present when dogs are infected but clinically healthy. This determines that the dog is the main infection focus for sandflies, and therefore for other dogs, other animals, and even humans. A serological diagnosis has been established as a very useful tool for the early detection of infection in dogs. This tool has proved effective for the detection of clinically healthy infected (CHI) dogs [4, 5].

*Leishmania infantum* can affect up to 70 different animal species, making it a severe zoonosis [6]. In humans, the disease is usually associated with a poor immune response and has been reported mainly in immunocompromised patients such as HIV and organ transplant patients [6]. In our region (Madrid Community), the incidence rate was 1.12 cases per 100,000 inhabitants and year according to studies carried out from 1999 to 2003 [7]. Later in 2012, this rate increased to 22.2 cases per 100,000 inhabitants [8]. To date, this epidemiological event is considered the largest outbreak of human leishmaniosis recorded in Europe. The outbreak started in 2009, affecting the municipalities of the southwestern region of Madrid (Leganés, Getafe, Fuenlabrada, and Humanes de Madrid), and although in remission, it remains active today. In the epidemiological season from the second semester of 2009 to the first semester of 2010, there was a significant increase in the number of cases that rose from 9 to 27, continuing with 173 in 2011, 206 in 2012, 64 in 2013, and 113 in 2014, and thereafter declining gradually to 84 in 2015, 75 in 2016, 78 in 2017, 50 in 2018, and 55 cases in 2019 [9]. It should be noted that the hare was confirmed as the main reservoir for the Madrid outbreak [10, 11].

In the Madrid Community, a surveillance system for this disease in stray dogs was implemented in 1996 [12], and later extended to cats, other potential reservoirs, and even sandflies [11, 13, 14]. The Canine Leishmaniosis Surveillance Plan in the Madrid region is based on monitoring the prevalence of *L. infantum* infection over time and detecting associated risk factors. For this, antibodies are determined in stray dogs by means of a test in April and another one in November on the basis that the activity of the vector is bimodal and linked to the weather, with mild temperatures in spring and autumn favouring the biology of the sandfly. This means that dogs infected in autumn could be detected in April and those infected in early summer could be recorded in November [12]. This surveillance system guided the search and detection of new reservoirs (e.g., hares and rabbits) in the human outbreak of the disease in the Madrid Community as the incidence rate in the dog was between a surprising 1.6–2 percent while in humans it had increased dramatically [8, 10, 15, 16].

Following on from our previous study [12], the aim of the present survey was to focus on data for the period between 2007 and 2018 to calculate the seroprevalence of *L. infantum* in stray dogs in the Community of Madrid and assess the impacts of the epidemiological variables sex, breed, age, clinical signs, and living or not in the human leishmaniasis outbreak area.

## Methods

### Study area

The present study was carried out in the Madrid Community (central Spain), whose altitude varies from 491 to 2400 m. Vegetation is also highly variable, with wooded areas of deciduous trees, pine forests, and scrub areas. The climate is of the Mediterranean-continental type with cold winters and hot, dry summers. Rains appear in spring and autumn and temperatures at this time are mild, around 20 °C in spring and 15 °C in autumn. However, of note is a significant increase in temperatures and a relative decrease in rainfall detected in the past 30 years [17–19].

### Study design

Based on vector phenology and climatology, two samplings were scheduled each year, one in spring (April–May) and one in autumn (November). The sample of dogs included in each period ranged from 100 to 150 dogs.

A total of 17 shelters were included in the present study. Each dog was subjected to the same protocol, which consisted of the collection of data regarding age, sex, breed, and clinical signs. Dog’s ages were estimated according to several factors (e.g., body condition, external appearance, development stage of genitals, and dentition), establishing four age groups (< 1 year, 1–3 years, 3–7 years and > 7 years). We established five size groups: x-small (< 6 kg), small (6–14.9 kg), medium (15–24.9 kg), large (25–39.9 kg), and x-large (≥ 40 kg). The municipality of capture of the animal was also recorded to examine the seroprevalence of *L. infantum* in relation to the whether or not the dogs were living in the area of the outbreak of human leishmaniosis (Getafe, Fuenlabrada, Leganés, and Humanes de Madrid).

### Samples and laboratory diagnosis

Blood and faeces samples were also collected from the dogs included in our study. Samples were kept at 4 °C until processed at the laboratory.

Specific antibodies to *L. infantum* were detecting using the indirect immunofluorescence antibody test (IFAT) against in-house cultured promastigotes. The IFAT for anti-*Leishmania*-specific immunoglobulin G (IgG) antibodies was performed as described previously using a cut-off ≥ 1:100 to define seropositivity [20].

For coprological analysis, we used the modified Telemann sedimentation method plus merthiolate-iodine-formalin staining followed by examination under a light microscopy [21].

### Statistical analysis

Seroprevalence was defined as the percentage of sample testing positive for antibodies to *L. infantum*. The chi-square test was used to assess associations between *L. infantum* seroprevalence and age, sex, breed, clinical signs compatible or not with infection, the presence of intestinal parasites, and belonging or not to the area of the human leishmaniosis outbreak. Significance was set at *p* ≤ 0.05.

## Results

**Fig. 1:**
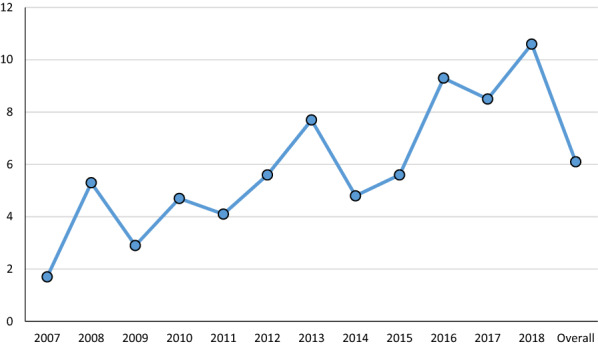
Overall seroprevalence of *Leishmania infantum* infection from 2007 to 2018

For a total of 3225 dogs included in this study, the overall seroprevalence of *L. infantum* was 6.1% (198/3225). Over the 12-year study period, seroprevalence has varied significantly from 1.7% recorded in 2007 to 10.6% in 2018 (*χ*^2^ = 34.5; *df* = 11; *p* < 0.001). As illustrated in Fig. 1, lowest seroprevalences were observed in 2007 (1.7%; 4/237), 2009 (2.9%; 6/209), 2010 (4.7%; 10/212), 2011 (4.1%; 9/217), and 2014 (4.8%; 15/310); intermediate seroprevalences in 2012 and 2015, both at 5.6% (17/305 and 17/303 respectively), and higher rates in 2016 (9.3%; 29/312), 2017 (8.5%; 28/329), and 2018 (10.6%; 28/265).

When seroprevalences were compared by season, no significant differences were observed between autumn with spring. These results were 5.5% (89/1,620) for the whole of the autumn compared to 6.8% (109/1605) recorded in the set of data for spring (*χ*^2^ = 2.35; *df* = 1; *p* = 0.125). In addition, no significant seroprevalence differences were observed between seasons in each year, nor when comparing autumns across the 12 years observed (*χ*^2^ = 17.6; *df* = 11; *p* = 0.091). In contrast, we did find significant differences between all springs (*χ*^2^ = 22.5; *df* = 11; *p* = 0.020), with the higher rates observed in spring 2017 (10.7%; 18/169) and 2018 (11.43%; 4/123) and the lowest in spring 2007 (2.4%; 3/124).

When we examined dog-related factors, seroprevalences recorded were similar at 6.4% for males (112/1750) and 5.7% for females (82/1437) (*χ*^2^ = 0.664; *df* = 1; *p* = 0.415); lower for puppies (< 1 year) (1.2%; 6/483) and dogs aged 1–3 years (4.9%; 84/1714) versus those aged 3–7 years (10.3%; 84/814) or > 7 years (12.6%; 23/183) (*χ*^2^ = 62.198; *df* = 3; *p* < 0.001); and higher for large (10.2%; 40/394) and x-large (12.2%; 10/82) versus medium (5.3%; 30/561), small (3.3%; 3/90) or x-small (2.3%; 1/44) dogs (*χ*^2^ = 14.745; *df* = 4; *p* = 0.005). Differences were also observed by breed: seroprevalence was 7.4% (94/1276) in pure breeds, while in mixed breeds it was 5.3% (103/1926) (*χ*^2^ = 5.206; *df* = 1; *p* = 0.023) and was significantly higher in dangerous breeds (breed-specific legislation) (10.8%; 27/249) and Dobermans (33.3%; 2/6) (*χ*^2^ = 7.493; *df* = 1; *p* = 0.006).

**Fig. 2:**
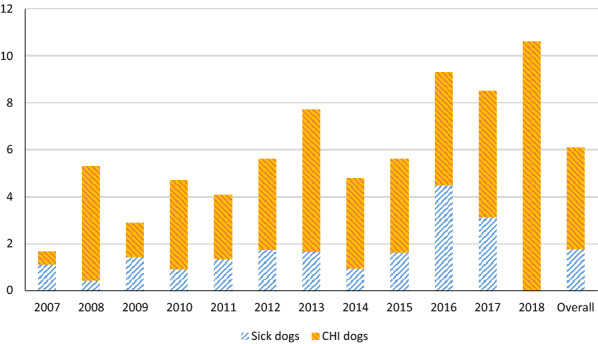
Seroprevalence of *Leishmania infantum* infection in sick and clinically healthy infected dogs

Clinical signs commonly observed in dogs were cutaneous lesions (alopecia, ulcers, hyperkeratosis, exfoliative dermatitis), lymphadenomegaly, diarrhoea, and lameness. The proportion of dogs with clinical signs compatible with leishmaniosis was 5.9% (176/2990). *Leishmania infantum* seroprevalence was 26.8% (53/198) in sick dogs and 64.6% (128 of 198) in CHI dogs (*χ*^2^ = 190.356; *df* = 1; *p* =  < 0.001) (Fig. 2).

**Fig. 3:**
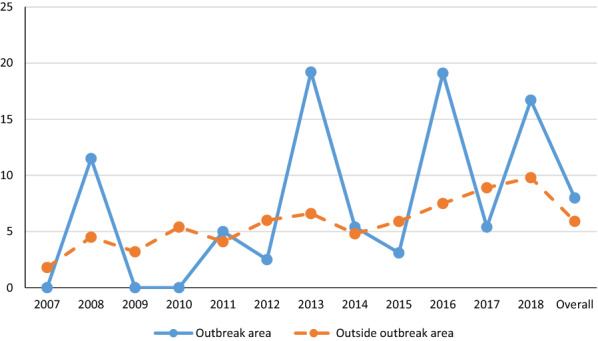
Seroprevalence of *Leishmania infantum* infection inside and outside of the outbreak area

No significant differences were observed according to geographical area (*χ*^2^ = 1.690; *df* = 3; *p* = 0.639), healthcare area (*χ*^2^ = 14.907; *df* = 8; *p* = 0.061), or outbreak area of human leishmaniosis (*χ*^2^ = 2.428; *df* = 1; *p* = 0.119). Hence, an overall seroprevalence of 8% (29/363) was observed inside the outbreak area and one of 5.9% (169/2862) outside the outbreak area (Fig. 3). Notwithstanding, over the 12-year study period, significant differences in this last variable emerged in 2013 (*χ*^2^ = 6.07; *df* = 1; *p* = 0.02) and 2016 (*χ*^2^ = 288.78; *df* = 1; *p* = 0.011), with higher seroprevalences recorded inside (2013: 19.2%, 5/26 versus 6.6%, 18/274; 2016: 19.1%, 9/47 versus 7.5%, 20/265) than outside the outbreak areas. Outside the human outbreak area, the seroprevalence of *L. infantum* has remained more or less stable, although more recently a significant increase was observed: 2017 (8.9%; 26/ 292) and 2018 (9.8%; 23/235) (*χ*^2^ = 24.224; *df* = 11; *p* = 0.012). Within the outbreak area, seroprevalence has been variable over the 12 years, as depicted in Fig. 3 (*χ*^2^ = 25.441; *df* = 11; *p* = 0.008).

From the 3225 dogs included in this study, 2114 stool samples were obtained, of which 492 had one or more intestinal parasites (23.3%). The intestinal parasites detected in order of prevalence were *Giardia duodenalis* (15.3%; *n* = 324), *Toxascaris leonina* (3.3%; *n* = 69), *Cystoisospora* spp. (2.6%; *n* = 54), *Toxocara canis* (2.2%; *n* = 46), Fam. Ancylostomatidae (2%; *n* = 42), *Trichuris vulpis* (1.2%; *n* = 26), Fam Taenidae (0.6%; *n* = 12), and *Dipylidium caninum* (0.05%; *n* = 1). Of 16 dogs out of 2990 dogs presenting with diarrhoea, however, none tested positive for *L. infantum* infection and only 6 had intestinal parasites. The seroprevalence observed among parasitized dogs was 4.5% (22/492) and that of non-parasitized dogs was 6.7% (109/1622), which was not significantly different (*χ*^2^ = 3.283; *df* = 1; *p* = 0.07).

## Discussion

The overall seroprevalence of *L. infantum* in this study was 6.1%, slightly lower than that observed (7.8%) in our similar study for the period 1996–2006 in stray dogs in the Madrid Community [12]. This seroprevalence is, nevertheless, slightly higher than that observed in other studies carried out in stray dogs in the Madrid region (4.7%; 5.4%) [22, 23]. If we compare our data (stray dogs) with those recorded previously in owned dogs, the seroprevalence of *L. infantum* infection is significantly lower in the latter (1.2–2.1%) [15]. These results are in agreement with those reported by other authors, who suggest that this difference in seroprevalence may be explained by stray dogs spending longer periods outside along with a lack of preventive measures against sandflies (e.g., use of repellents and insecticides) [24, 25].

It should also be considered that while mean seroprevalence for our 12-year study period (2007–2018) was 6.1%, over this period, seroprevalence values have been highly variable, the lowest value (1.7%) being recorded in 2007 and the highest in 2016 (9.3%) and 2018 (10.6%). Similar variability in seroprevalence data was observed over 10 years (1996–2006) in a study by Miró et al. (2007) [12]. Our present results, however, point to an increasing trend in seroprevalence in the more recent years. In a questionnaire-based survey addressing the clinical management of canine leishmaniosis in veterinary clinics across Spain, 34.9% of participating veterinarians felt that the incidence of *L. infantum* infection could be rising while 50.2% thought that incidence rates were currently fairly stable [26]. Similarly, other studies carried out in the Madrid region have not detected an increase in the incidence of canine leishmaniosis [15].

The main risk factor affecting the incidence of canine leishmaniosis is the presence of sandflies infected with *L. infantum* [27]. In addition, factors associated with the vector such as the climate, presence of organic matter, and reservoirs can explain geographical differences in prevalence and these may be related to human activities [28–30], as observed in the outbreak of human leishmaniosis in Madrid [31]. Our present data suggest that in this outbreak area, seroprevalence has been highly variable throughout the study period, giving rise to four peaks in 2008 (11.5%), 2013 (19.2%), 2016 (19.2%), and 2018 (16.7%) interspersed with years in which there was no case as in 2007 and 2009, at which time the outbreak was declared in the Madrid Community [8, 31]. Outside the outbreak area, *L. infantum* seroprevalence has been more stable across these study years, although higher seroprevalences were noted in 2017 and 2018 in line with the increasing trend produced in the Madrid Community. When comparing these areas, overall seroprevalences were not significantly different (*p* = 0.119): 8.0% (29/363) in the outbreak area and 5.9% (169 of 2862) outside this area. However, differences between the zones emerged in 2013 and 2106 in that the seroprevalence was significantly higher in stray dogs inhabiting the outbreak area. Accordingly, these results identify the outbreak area as a risk zone where sandflies remain infected. While the dog as a reservoir is an important component of the life cycle of the parasite, hares have been confirmed as a main reservoir in the outbreak of human leishmaniosis [10]. For all these reasons, we believe that stray dogs are a good indicator of the presence of *L. infantum* infection. In effect, the LeishSP implemented by the Madrid Community is an excellent early warning system for the surveillance of this disease. Moreover, given that the number of cases in humans has not reflected the changes observed in the dog seroprevalence map, we may also conclude that human infection in the outbreak area is in remission. We should, nonetheless, warn against lowering our guard as the parasite continues to circulate in this area.

Our seroprevalence rates of *L. infantum* compared between the springs (6.8%) and autumns (5.5%) of the same years are consistent with recent rates reported for owned dogs [32]. However, when seroprevalences obtained in each season of the year are compared, the lowest seroprevalence occurs in the autumn of 2007 (0.9%) and the highest in the spring of 2018 (11.4%). Other authors have also reported higher seroprevalences in spring versus autumn [12]. The reason for this could lie in a larger proportion of dogs infected in late summer and/or early autumn and these infections being detected in the following spring as anti-*L. infantum* antibodies appear between 90 and 120 days post-infection [33]. In experimental infections, this period of incubation may be as long as 180 days [34]. In addition, the detection of a greater number of seropositive animals in spring may be because the weather conditions for sandflies in the Madrid Community are more favourable over a longer period as autumn conditions may continue into November, thus allowing for the transmission of *L. infantum* [18, 19, 35].

If we compare our seroprevalence data with those reported for other Spanish provinces, these are also variable [23, 36]. However, few studies have been conducted in stray dogs and these are not always comparable in terms of the different diagnostic techniques used, size and origin of samples, and study duration, among other factors. For example, reports exist of seroprevalences in owned dogs of 34.6% in Malaga, 33.1% in Lleida, 24.6% in Huesca, 22% in Alicante province, and 4.74 to 31% in the Balearic Islands [23, 37]. In contrast, in the north and northwest of Spain, respectively, seroprevalences of 0–5% and 3.7–10.8% have been reported [32, 38, 39]. In stray dogs, higher seroprevalences were observed in the same area: 2–4.7% in the north and 35.6% in Orense [23]. In a cross-sectional serological survey conducted from 2011 to 2016, four risk areas of *L. infantum* infection in dogs in general were identified: (1) non-endemic or low risk (0%), (2) hypoendemic or medium risk (0.1–7%), (3) intermediate-high risk (8–16%), and (4) high-risk or hyperendemic (> 16%) [23]. While our overall seroprevalence was 6.1%, it should be considered that in more recent years a higher seroprevalence was recorded (8.5–10.6%), consistent with the increasing trend described for central Spain by Gálvez et al. [23].

As discussed above, we also need to consider epidemiological factors related to the main host (e.g., age, sex, breed, immunological status, habitat, genetics) [40]. Our data revealed no significant seroprevalence differences related to sex (*p* = 0.415), in agreement with the findings of others [12, 25, 36, 41, 42]. However, some authors have detected a higher seroprevalence in male dogs possibly because of their roaming behaviour [43, 44]. In 2002, Travi et al. described that adult male hamster presented with more extensive cutaneous lesions due to *L. infantum* infection than prepubertal males and so suggested testosterone may be involved in the difference observed between sexes [45].

For the groups of dogs in our study aged < 3 years, seroprevalence was lower than in older dogs indicating a unimodal prevalence distribution in agreement with prior work. Dogs > 4 years old showed the greatest probability of infection, explained by their longer exposure to sandflies [46]. Other authors have reported a bimodal pattern with a higher seroprevalence in animals < 3–4 years or > 7–8 years old [12, 25]. This elevated seroprevalence in young dogs could be related to a genetic predisposition or immune system immaturity [43].

Significant seroprevalence differences were also observed between purebred (7.4%) and mixed-breed dogs (5.3%). While this difference was not detected in our prior study [12, 25, 44], Cortés et al. observed a lower seroprevalence in mongrel dogs [47]. Likewise, in studies carried out in Brazil, pure breeds (Poodle, Pincher, and Pit Bull) were also found to show a higher seroprevalence than mixed breeds [48]. In our study, the dog breeds with the highest seroprevalences were a group of dangerous breeds (10.8%) and Dobermans (33.3%). Other authors have also detected higher seroprevalences in breeds such as Boxer, Doberman, German Shepherd, Rottweiler, and Cocker Spaniel [37, 43, 49]. In contrast, the Ibizan Hound is more resistant to infection as it seems that this breed or mixes with this breed elicits an adequate cellular response against *L. infantum* infection. A different seroprevalence associated with breed could be attributed to a genetic factor making some breeds more resistant to the pathogenic actions of the parasite [50]. More studies are needed to determine the genetic factors related to canine leishmaniosis.

As dog size is linked to breed, we observed a significantly higher seroprevalence in large (10.2%) and x-large dogs (12.2%) than in medium (5.3%), small (3.3%), and x-small (2.3%) ones, in line with published data [38, 44, 51]. A greater seroprevalence in larger dogs may reflect a greater body surface for sandflies to feed on, and therefore a greater likelihood of infection, as observed with other parasite diseases such as canine thelaziosis [52]. Furthermore, large breed dogs are often used as guard dogs and spend more time outdoors; consequently, these dogs are more susceptible to the bites of female sandflies [25, 47].

The proportion of sick dogs with clinical manifestations compatible with canine leishmaniosis in our study was 5.9% (176/2990). The most frequent clinical signs were cutaneous lesions (alopecia, ulcers, hyperkeratosis, exfoliative dermatitis), lymphadenomegaly, and lameness, as described by others [2]. In effect, these clinical signs are commonly observed by veterinarians [26].

*Leishmania infantum* seroprevalences were 26.8% (53/198) in sick dogs and 64.6% (128/198) in clinically healthy infected dogs, in line with previous reports [12, 29]. This significantly higher seroprevalence in CHI dogs highlights the importance of an early diagnosis in dogs without clinical signs, as these could be a risk for other animals and humans [34].

Dogs included in the present study were stray, so they often had clinical signs associated with this lifestyle (e.g., poor body condition and gastrointestinal disorders). So, it is difficult to associate some general clinical signs with *L. infantum* infection, such as diarrhoea, which is rarely observed in canine leishmaniosis [53]. In our study, this clinical sign was observed in only 0.5% of dogs (16/2990). None were infected with *L. infantum*, but six had intestinal parasites as a possible cause of diarrhoea.

In addition, no association was found here between the presence of intestinal parasites and the seroprevalence of *L. infantum*; the prevalence of parasites among seropositive dogs was 4.5% compared with 6.7% for seronegative dogs. In other words, there is no greater risk of a dog getting leishmaniosis if it has intestinal parasites, although this situation could indicate immunocompromise. Our results are similar to those found in earlier studies in which we noted no significant association between intestinal parasites and *L. infantum* [12].

## Conclusions

Our findings highlight the important role of stray dogs as sentinels of *L. infantum* infection and confirm the effectiveness of the LeishSP implemented by the Madrid Community as an early warning strategy for human and animal leishmaniosis.

## Data Availability

All data generated or analysed during this study are included in this published article.

## Abbreviations

CHI: Clinically healthy infected
HIV: Human immunodeficiency virus
HL: Human leishmaniosis
Ig: Immunoglobulin
IFAT: Immunofluorescence antibody test
Kg: Kilogram
LeishSP: Leishmaniosis Surveillance Program
